# Selected Electrochemical Properties of 4,4’-((1E,1’E)-((1,2,4-Thiadiazole-3,5-diyl)bis(azaneylylidene))bis(methaneylylidene))bis(*N*,*N*-di-p-tolylaniline) towards Perovskite Solar Cells with 14.4% Efficiency

**DOI:** 10.3390/ma13112440

**Published:** 2020-05-27

**Authors:** Krzysztof Artur Bogdanowicz, Beata Jewłoszewicz, Agnieszka Iwan, Karolina Dysz, Wojciech Przybyl, Adam Januszko, Monika Marzec, Kacper Cichy, Konrad Świerczek, Ladislav Kavan, Markéta Zukalová, Vojtech Nadazdy, Riyas Subair, Eva Majkova, Matej Micusik, Maria Omastova, Mehmet Derya Özeren, Katalin Kamarás, Do Yeon Heo, Soo Young Kim

**Affiliations:** 1 Military Institute of Engineer Technology, Obornicka 136 Str., 50-961 Wroclaw, Poland; jewloszewicz@witi.wroc.pl (B.J.); dysz@witi.wroc.pl (K.D.); przybyl@witi.wroc.pl (W.P.); januszko@witi.wroc.pl (A.J.); 2 Institute of Physics, Jagiellonian University, 11, 30-348 Krakow, Poland; Monika.Marzec@uj.edu.pl; 3 Faculty of Energy and Fuels, AGH University of Science and Technology. A. Mickiewicza 30, 30-059 Krakow, Poland; Cichykac@agh.edu.pl (K.C.); xi@agh.edu.pl (K.Ś.); 4 AGH Centre of Energy, AGH University of Science and Technology, ul. Czarnowiejska 36, 30-054 Krakow, Poland; 5 Department of Electrochemical Materials, J. Heyrovsky Institute of Physical Chemistry, Czech Academic Science, Dolejskova 3, 18223 Prague, Czech Republic; ladislav.kavan@jh-inst.cas.cz (L.K.); marketa.zukalova@jh-inst.cas.cz (M.Z.); 6 Institute of Physics, Slovak Academy of Science, Dubravska cesta, 9, 845 11 Bratislava, Slovakia; Vojtech.Nadazdy@savba.sk (V.N.); Riyas.Subair@savba.sk (R.S.); eva.majkova@savba.sk (E.M.); 7 Centre for Advanced Material Application, Slovak Academy of Sciences, Dúbravská cesta, 9, 845 11 Bratislava, Slovakia; 8 Polymer Institute, Slovak Academy of Science, Dubravska cesta, 9, 845 41 Bratislava, Slovakia; matej.micusik@savba.sk (M.M.); maria.omastova@savba.sk (M.O.); 9 Institute for Solid State Physics and Optics, Wigner Research Centre for Physics, Hungarian Academy of Sciences, Konkoly Thege u. 29-33, 1121 Budapest, Hungary; mehmet.ozeren@wigner.hu (M.D.Ö.); kamaras.katalin@wigner.hu (K.K.); 10 Department of Materials Science and Engineering, Korea University 145, Anam-ro Seongbuk-gu, Seoul 02841, Korea; doyoun0312@naver.com (D.Y.H.); sooyoungkim@korea.ac.kr (S.Y.K.)

**Keywords:** imines, azomethines, perovskite solar cells, HTM, electrochemical impedance spectroscopy, thermographic camera

## Abstract

Planar perovskite solar cells were fabricated on F-doped SnO_2_ (FTO) coated glass substrates, with 4,4’-((1E,1’E)-((1,2,4-thiadiazole-3,5-diyl)bis(azaneylylidene))bis(methaneylylidene))bis(*N*,*N*-di-p-tolylaniline) (bTAThDaz) as hole transport material. This imine was synthesized in one step reaction, starting from commercially available and relatively inexpensive reagents. Electrochemical, optical, electrical, thermal and structural studies including thermal images and current-voltage measurements of the full solar cell devices characterize the imine in details. HOMO-LUMO of bTAThDaz were investigated by cyclic voltammetry (CV) and energy-resolved electrochemical impedance spectroscopy (ER-EIS) and were found at −5.19 eV and −2.52 eV (CV) and at −5.5 eV and −2.3 eV (ER-EIS). The imine exhibited 5% weight loss at 156 °C. The electrical behavior and photovoltaic performance of the perovskite solar cell was examined for FTO/TiO_2_/perovskite/bTAThDaz/Ag device architecture. Constructed devices exhibited good time and air stability together with quite small effect of hysteresis. The observed solar conversion efficiency was 14.4%.

## 1. Introduction

Perovskite solar cells (PSC) are attractive due to their record solar conversion efficiency near 25%, approaching already the efficiencies of the best solid-state photovoltaics (Si, GaAs, CIGS, CdTe) [1,2]. The PSC is sometimes called a ‘young sister’ of dye-sensitized solar cell [3,4] to highlight their common principles: The generic perovskite, CH_3_NH_3_PbI_3_ was actually disclosed for the first time (in 2009) using a liquid-junction photoelectrochemical device [1,3,4]. Yet, the electrochemical approaches are still rather scarce in PSC research [4]. In the regular (and so far the most efficient) n-i-p architecture of PSC, the oxide-based electron-selective layer (usually TiO_2_) provides a negative electrical contact to the perovskite photo-absorber. The second (positive) electrical contact is fabricated from hole transporting material (HTM), like CuSCN [5] or *spiro*-OMeTAD, the latter being ubiquitously used in almost all highly-efficient PSCs [1]. 

Nevertheless, *spiro*-OMeTAD hole transporter also brings variety of problems. First, it requires oxidative doping, mostly employing lithium bis(trifluoromethanesulfonyl)imide, (LiTFSI). This introduces some stability issues, because LiTFSI is hygroscopic and brings moisture degrading to the perovskite. Also *spiro*-OMeTAD can degrade by diffusion of Au or Ag (from the positive terminal), reactions of iodide (from the perovskite), crystallization and photooxidation [1]. Last but not least, *spiro*-OMeTAD is also very expensive, actually dearer than gold or platinum [6,7]. These obstacles incentivize a search of alternative organic hole conductors, and this was the central motivation of our study.

The main disadvantage of new organic HTM materials, until now, is the fact that they are synthesized via cross-coupling reactions that require transition metal catalysts, inert reaction conditions and extensive product purification [6]. This makes perovskite solar cells expensive and difficult for scaling up production and application as building materials in photovoltaics (BIPV). Simplification and reduction of synthesis steps should be an asset for potential new HTM.

HTM for perovskite solar cells can be classified into three main groups [8]:

1. Organic HTMs including polymers (poly(3-hexylthiophene): P3HT, poly(3,4-ethylenedioxythiophene): PEDOT, crosslinked polymers) and small molecular materials (*spiro*-OMeTAD, pyrene-, thiophene-, carbazole-, porphyrin derivatives) based HTMs,
2. Inorganic HTMs combine nickel and copper-based HTMs (NiO, CuO, CuSCN, CuCrO_2_, CuGaO_2_), p-type based semiconductor HTMs (such as organometallic complexes, e.g., coper phthalocyanine or coper thiocyanate- based HTMs), and transition metal oxide based HTMs (MoO_3_, VO_x_, WO_x_),
3. Carbonaceous based HTMs (graphene oxide based HTMs).

Organic HTMs use small molecules based on triphenylamine (TPA) [6,9,10,11,12,13,14,15,16,17,18,19]. In [17] the authors investigated an amide-based small-molecule with TPA units (EDOT-Amide-TPA). For perovskite solar cell architecture such as FTO/SnO_2_/FA_0.83_Cs_0.17_Pb(I_0.83_Br_0.17_)_3_/HTM/Au (FA = formamidinium) the power conversion efficiency (PCE) equal to 20.3% and fill factor (FF) 77% were obtained. Moreover, the investigation of ultraviolet-visible (UV–Vis) absorption spectra of EDOT-Amide-TPA films in a function of LiTFSI addition showed that the formation of the oxidized species detected in the range of 700–800 nm and a red-shift in the absorption maximum (400–440 nm) of the original compounds were due to the coordination of the lithium-ions with the amide bond.

In another work [16] the authors investigated two new small molecules containing TPA units together with thiophene and phenylene (Z25) or thiophene and vinylene-thiophene (Z26) units as core. The compound Z26 exhibited the PCE = 20.1% (FF = 75%), while the compound Z25 showed lower values (PCE= 16.9%, FF = 64%).

Petrus et al. [15] proposed interesting small azomethine molecules: thiophene or benzenothiazolo-units as core and two TPA units at the end of molecules, for application in organic solar cells. They conducted an in-depth investigation of all crucial parameters for application in organic photovoltaics. The best device was obtained by using the donor to acceptor ratio equal to 1:5 (azomethine:PCBM) with layer thickness of ∼70 nm. For optimized device PCE of 2.17% was achieved. 

Another work of Petrus et al. [6] used azomethine-based small molecule with TPA units (EDOT-OMeTPA) which provided PCE of 11.0% for the HTM layer thickness of ca. 40 nm. A control device with spiro-OMeTAD provided PCE = 11.9% in the same work [6].

In our previous work [20] we investigated perovskite solar cells with HTMs based on polyazomethines with carbazole and thiophene units. For the device with the architecture FTO/TiO_2_/TiO_2_/perovskite/polyazomethine/Au, the power conversion efficiency of about 8% was obtained. Moreover, stability test proved the prolonged effects of polyazomethines on perovskite absorber.

Herein, we report the selected electrochemical properties of a symmetrical imine, 4,4’-((1E,1’E)-((1,2,4-thiadiazole-3,5-diyl)bis(azaneylylidene))bis(methaneylylidene))bis(*N*,*N*-di-p-tolylaniline) (abbreviated bTAThDaz) with the aim of its use in perovskite solar cells as hole transporting material (HTM). Our imine was characterised by cyclic voltammetry and energy-resolved electrochemical impedance spectroscopy to assess its applicability in perovskite solar cell construction. Morphology, composition and structure of the obtained layers were investigated by scanning electron microscopy (SEM) together with X-ray photoelectron spectroscopy (XPS) and X-ray diffraction (XRD). Thermal properties of the investigated imine were checked by thermogravimetric analyses in two different atmospheres, synthetic air and Ar. FTIR spectra were measured in wide temperature range to investigate possible inter- or intramolecular interactions in our imine. The thermographic camera was used to detect the location of defects in the fabricated devices and the electrical behavior of the investigated imine. To check the crucial electrical properties, the hysteresis effect in the investigated compound, the electrical and thermal response to forward and reverse bias was also studied. Finally perovskite solar cells with our imine as HTM was constructed and compared with a control device with *spiro*-OMeTAD. New materials, that can replace the currently used HTMs such as *spiro*-OMeTAD represent the current trends in organic and perovskite solar cells, taking into consideration price and some synthesis steps of the currently used compounds. Here we present, for the first time, the perovskite solar cells with imine bTAThDaz as HTM with efficiency exceeding 14%.

## 2. Experimental Section

The electronic structure of the imine thin film was measured by energy-resolved electrochemical impedance spectroscopy (ER-EIS) [21,22]. The imine and *spiro*-OMeTAD films were spun coated on indium tin oxide (ITO) substrate in a glove box under N_2_. The microcells for electrochemical measurements was made of plastic cone and consisted of disc working electrode (area of 12 mm^2^), Ag/AgCl reference electrode and Pt wire as counter electrode. The measurements were controlled by potentiostat (home-made) and performed in 0.1 M TBAPF_6_ (Sigma Aldrich, Bratislava, Slovakia) solution in anhydrous acetonitrile. To calculate the local vacuum level a potential of −4.66 eV for Ag/AgCl reference electrode energy vs. vacuum was assumed. The impedance measurements were performed with AC harmonic voltage signal frequency equal 0.5 Hz, the rms value of 100 mV, and the sweep rate of the DC voltage ramp 10 mV∙s^−1^. The results of impedance experiment were controlled by an impedance/gain-phase analyzer, Solartron analytical, model 1260 (Ametek, Berwyn, IL, USA). 

Other electrochemical measurements were carried out, as described elsewhere [23], using a Metrohm Autolab PGSTAT M204 potentiostat (Barendrecht, The Netherland), glassy carbon electrode (diam. 2 mm) and a platinum rod and Ag/AgCl. As reference redox system ferrocene (Fc) was used. Cyclic voltammetry experiments were conducted in a standard one-compartment cell, in acetonitrile (≥ 99,9%, Honeywell, Charlotte, NC, USA), under argon. 0.2 M Bu_4_NPF_6_ (Alfa Aesar, 99%, Haverhill, MA, USA) was used as the supporting electrolyte. The concentration of bTAThDaz compounds was equal to 1.0 × 10^−6^ mol∙dm^−3^. The deaeration of the solution was achieved by argon bubbling through the solution for about 15 min prior to the measurement. All electrochemical experiments were carried out at ambient temperature and pressure.

The optical (UV-Vis) spectra were measured using a PerkinElmer Lambda 19 UV-VIS-NIR spectrophotometer (PerkinElmer, Waltham, MA, USA), either in chlorobenzene solution (quartz optical cell, 1 cm length) or on thin films supported by quartz plates. To fabricate high-quality films on quarts, the solvents’-purified substrate (see below) was subjected to additional cleaning in plasma using the piezo-brush PZ2 (Relyon plasma, GmbH, Regensburg, Germany). 

Powder X-ray diffraction (XRD) was studied on a Bruker D8 Advance diffractometer (Bruker, Billerica, MA, USA) using CuKα radiation. 

Scanning electron microscopy (SEM) images were obtained by a Hitachi S-4800 microscope (Hitachi, Marunouchi, Chiyoda-ku, Tokyo, Japan).

The conductivity was measured by using the van der Pauw contact configuration of quartz-supported thin film. The gold contacts were deposited in the corners of the square film samples. Two of the contacts served as current input/output; the other two contacts were used for voltage measurement. A Keithley 6430 current source (Tektronix Inc., Solon, OH, USA) and two Keithley 6514 electrometers (Tektronix Inc., Solon, OH, USA) monitoring potentials at voltage contacts were used; the resulting voltage drop was measured by connecting a Keithley 2182A nanovoltmeter to the analogue low-impedance outputs of the electrometers. The conductivity across the film was measured by two-points probe using the Au-supported thin film. 

XPS spectra were recorded using a Thermo Scientific K-Alpha XPS system (K-Alpha 2010, Thermo Fisher Scientific Inc., East Grinstead, UK) equipped with a microfocused, monochromatic Al Kα X-ray source (1486.68 eV). An X-ray beam of 400 µm size was used at 6 mA × 12 kV. The spectra were acquired in the constant analyser energy mode with pass energy of 200 eV for the survey. Narrow regions were collected with pass energy of 50 eV. The Thermo Scientific Avantage software, version 5.9915 (Thermo Fisher Scientific Inc., East Grinstead, UK), was used for data acquisition and processing. Spectral calibration was achieved by using the automated calibration routine and the internal Au, Ag and Cu standards supplied with the K-Alpha system. The surface compositions (in at%) were determined by considering the integrated peak areas of the detected atoms and the respective sensitivity factors. The fractional concentration of a particular element A was computed using:

(1) $$\%\;A=\frac{I_{A}/s_{A}}{\sum \left(I_{n}/s_{n}\right)}\times 100\%$$

where *I* and *s* are the integrated peak areas and the Scofield sensitivity factors corrected for the analyzer transmission, respectively. (The subscripts ‘*A*’ or ‘*n*’ mean the element *A* or any element detected in the surface, respectively).

Differential scanning calorimetry (DSC) measurements were carried out using a Perkin Elmer DSC8000 calorimeter (PerkinElmer Inc., Waltham, MA, USA). The temperature was calibrated on the onsets of melting points of water and indium. The sample was hermetically sealed in aluminium pans of 30 µL. Measurements were done during cooling/heating ramp of 10 °C/min under nitrogen atmosphere. 

Thermal behaviour was investigated using a thermographic camera (VIGOcam v50, VIGO System S.A, Ożarów Mazowiecki, Poland) while applying bias voltage between 0 and 10 V and using a multichannel potentiostat-galvanostat (PGStat Autolab M101, Metrohm, The Netherlands). The experiment was designed to apply voltage in a programmed way as follows: the potential was applied in range from the 0 V to 10 V with 0.5 V step between different values during three minutes for each voltage. The current response was recorded during this three minute-intervals and each step was separated with 10 s window, when the IR image was collected, while the current was still passing through the sample (see Figure S1). Both camera and power source were digitally controlled. For this experiment the samples were prepared by spin-coating technique using 5000 rpm/20 s for two solutions 15 mg/mL w/w and 10 mg/mL giving ca. 40 nm and 20 nm, respectively.

The Fourier transform infrared (FTIR) spectra of the imine in the region of 4000–400 cm^−1^ were measured at 4 cm^−1^ resolution with accumulation of 256 scans on a Bruker Tensor 37 spectrometer (Bruker Optics, Ettlingen, Germany) with an MCT detector using the KBr pellet technique.

Thermogravimetric (TGA) measurements were performed on TA Q5000 IR thermobalance (New Castle, U.S) using Pt holders. Behaviour of the imine was examined under two different atmospheres of synthetic air and Ar (5N). The gas flow was 100 mL∙min^−1^, supplied directly in the vicinity of the studied sample. Each TGA test included heating in a temperature range of 30–800 °C with a rate of 10°∙min^−1^. 

### 2.1. Deposition of Thin-Film

FTO glass (TEC 15 from Greatcell Energy, 15 Ohm/sq, Greatcell Energy, Eleanora, QLD, Australia) was cleaned ultrasonically using a bath-type cleaner (Sonorex Digital 10P, Bandelin, 320W, 35 kHz, Bandelin electronic GmbH&Co.KG, Berlin, Germany) with (sequentially) isopropanol, ethanol and acetone (10 min treatment in each bath, Aldrich, Sigma-Aldrich Ltd., Prague, Czech Republic). Alternative substrates for thin-film deposition were optical quartz or microscope glass; they were cleaned in the same way. For conductivity measurements, the substrate was 90 nm gold layer which was vacuum-deposited on top of 20 nm Ti layer on glass. A freshly made Au/Ti/glass substrate was used as is, i.e., without cleaning.

A thin film of bTAThDaz was deposited from 70 mM chlorobenzene solution. The solution was carefully homogenized by ultrasonic treatment (bath-type cleaner as above) as long as the SEM images of the final film contained no visible particles (see Figure S2, exhibiting the typical morphology of a non-optimized film containing undissolved particles). The deposition was carried out by spin-coating method (Laurel Technologies Corp. spin-coater, Laurel Technologies Corp., North Wales, PA, USA) at 6000 rpm/20 s. The dynamic mode of operation was employed (the solution was dropped onto the substrate under rotation).

### 2.2. Construction of Perovskite Solar Cells with bTAThDaz as HTM

Etched FTO/glass (15 Ω∙sq^−1^) was cleaned with acetone, isopropyl alcohol, deionized water (15 min in each solvent). A compact TiO_2_ layer (cp-TiO_2_) was deposited onto FTO by spin-coating of 55 μL titanium diisopropoxide bis(acetylacetonate) (75wt% in isopropanol, Sigma-Aldrich, Saint Louis, MO, USA) dissolved in 1 mL 1-butanol (99.8%, Sigma-Aldrich, Saint Louis, MO, USA) at 1500 rpm for 30 s, which was dried at 120 °C for 10 min. A mesoporous TiO_2_ layer (ms-TiO_2_) was formed on the cp-TiO_2_ by spin-coating the diluted TiO_2_ paste (0.25 g∙mL^−1^) at 3000 rpm for 30 s. After the substrate was dried at 120 °C for 10 min, it was then sintered at 450 °C for 1 h. After UV-ozone treatment for 30 min to ensure good wettability, the substrate was moved to an N_2_-filled glove box. The perovskite solution was prepared by dissolving PbI_2_ (99.98%, Alfa Aesar, Ward Hill, MA, USA) and CH_3_NH_3_I (OMNISCIENCE) in 9:1 *N*,*N*-dimethylformamide (DMF, 99.8% anhydrous, Sigma-Aldrich) and dimethyl sulfoxide (DMSO, 99.9% anhydrous, Sigma-Aldrich, Saint Louis, MO, USA). The perovskite precursor solution (1.5 M CH_3_NH_3_PbI_3_) was spin coated on the substrate at 4000 rpm for 30 s, and 1 mL of toluene (99.8% anhydrous, Sigma-Aldrich, Saint Louis, MO, USA) was dropped on the rotating substrate in 10 s. The substrate was dried at 100 °C for 30 min. A *spiro*-OMeTAD and bTAThDaz solution were prepared by dissolving 0.059 M *spiro*-OMeTAD and bTAThDaz in each 1 mL chlorobenzene, to which 28.8 μL of 4-*tert*-butylpyridine and 17.5 μL of lithium bis(trifluoromethanesulfonyl)imide (LiTFSI) solution (720 mg/1 mL acetonitrile, Sigma-Aldrich, Saint Louis, USA) were added. The *spiro*-OMeTAD and bTAThDaz solution were spin-coated on the perovskite film at 3000 rpm for 30 s. Finally, silver (ITASCO, Seoul, Korea) was thermally evaporated. 

The current density–voltage (*J*–*V*) characteristics of the PSCs were measured using a Keithley 2614B semiconductor parameter analyser (Keithley, OH, USA). The measurement was conducted under AM 1.5G 100 mW∙cm^−2^ illumination using an 150 Watt xenon arc lamp solar simulator (HS Technologies, Seoul, Korea). All devices were measured by masking the active area with a thin mask (0.14 cm^2^). The *J*–*V* characteristics of all the devices were measured at a voltage scan rate of 0.1 V∙s^−1^.

## 3. Results and Discussion

### 3.1. Synthesis and Purification of bTAThDaz

**Figure 1:**
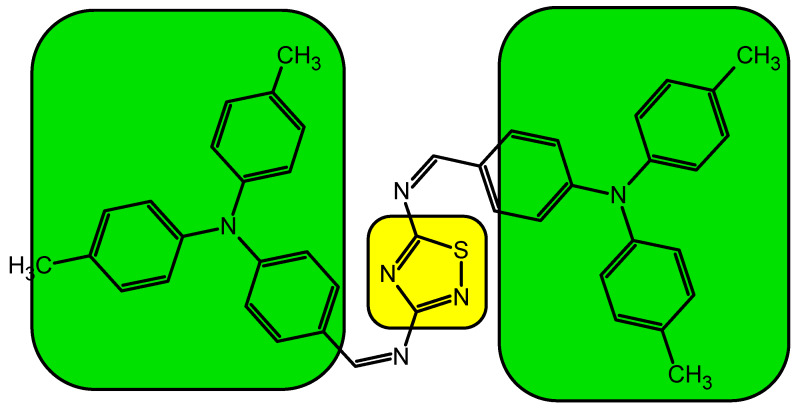
Chemical structure of the synthesized imine bTAThDaz.

The imine was synthesized in a one-step condensation reaction in solution between of 4-(di-p-tolylamino)benzaldehyde) and diamine such as 1,2,4-thiadiazole-3,5-diamine, where water was the main by-product (see Figure 1). Details about synthesis are presented in [24,25] and in Supplementary Materials. The fact that the synthesis path of this imine proposed by us was performed in a single step reaction followed by simple purification significantly lowers the production cost. The simplicity of the whole process allows solvent recovery, i.e., ethanol, acetone or *N*,*N*-dimethylacetamide (DMA). Moreover, in the process are not included any expensive catalysts or inorganic compounds and the main by-product – water does no harm to the environment and can be reused [26].

Special attention was paid to purification of the obtained imine by applying various solvents (ethanol, acetone), combined with crystallization from acetone-hexane mixture. The purification progress was monitored by thin layer chromatography (TLC) and proton nuclear magnetic resonance (^1^H NMR). After the reaction, the raw product contained unreacted aldehyde. The second purification, including recrystallization from acetone-hexane resulted in pure product. 

The symmetrical imine was characterized by XPS and XRD. Additional characteristics by ^1^H NMR and FTIR spectroscopy are presented in Supplementary Materials and in [24,25]. All the experimental data were consistent with the proposed structure. The XPS spectra of the investigated imine are depicted in Supplementary Materials (Figure S3), while concentrations and binding energies of the identified functional groups are listed in Table S1. Powder X-ray diffractogram of bTAthDaz showed amorphous nature of the investigated imine with a broad peak at 2Θ = 20° (Figure S4a, Supplementary Materials).

**Figure 2:**
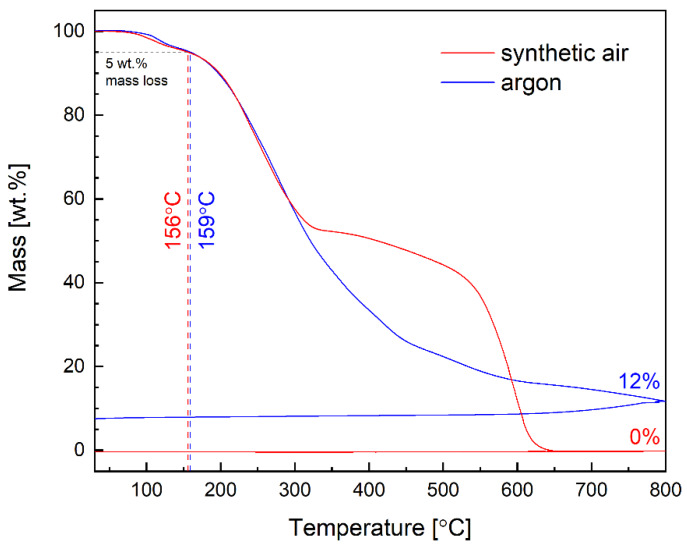
TGA curves of bTAThDaz recorded in air and argon atmospheres.

The thermogravimetric analysis (TGA) was performed in argon and synthetic air atmospheres with a heating rate of 10 °C/min, from room temperature up to 800 °C (Figure 2). 

Shape of the TGA curves of imine indicates two main reaction stages occurring in both atmospheres. Assuming 5 wt.% loss as a criterion for assessing thermal stability of the material, this values is reached at ca. 156–159 °C, being almost independent on the atmosphere. Consequently, it can be stated that TGA analysis proved good thermal stability of the material in both gasses. At higher temperatures, exceeding ca. 300 °C in this particular case, some differences can be observed between both recorded curves. Different behavior in 300–550 °C range seems to reflect different oxygen partial pressures, suggesting that further decomposition proceeds easier at low pO_2_. On the other hand, at the final stage of the process, above 600 °C, lack of oxygen in Ar causes some amount of carbonized residue to be present after whole TGA cycle, while in air, this residue is oxidized, with the sample being completely decomposed at ca. 650 °C (Figure 2). Obviously, good thermal stability of the considered material is crucial concerning usage in organic solar cells.

DSC thermogram registered at heating for bTAThDaz is presented in Figure S4b in Supplementary Materials. Because the studied compound is stable during cooling up to 100 °C, which is why only the interesting temperature range is presented. As can be seen DSC curve shows two very small anomalies at 35.1 °C and 53.8 °C with enthalpy changes of −0.32 J/g and −0.66 J/g, respectively which may be probably responsible for crystallization process in two stages.

**Figure 3:**
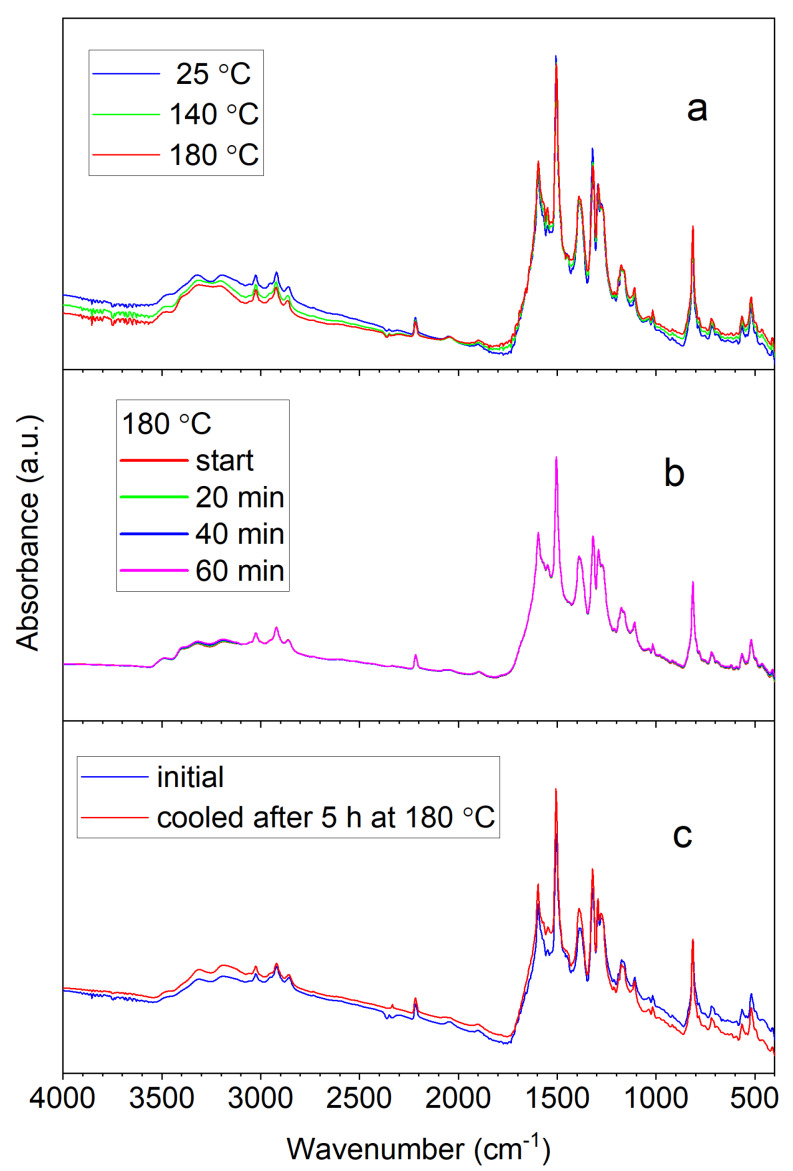
Temperature dependence of FTIR spectra of bTAThDaz (**a**), time dependence of the IR spectrum of bTAThDaz at 180 °C (**b**) and IR spectrum of bTAThDaz at 180 °C before and after annealing at 180 °C (**c**).

Infrared spectra and their assignment were reported in Ref. [25]. We followed the temperature dependence of the spectra in nitrogen atmosphere to see if any structural transitions occur prior to thermal degradation that would indicate the presence of intra- or intermolecular secondary bonds. The spectra did not change upon heating from room temperature to 180 °C and being kept at this temperature for 5 h (Figure 3 and Figure S5), showing that both the molecular and crystal structure are thermally stable. 

### 3.2. Optical and Electrochemical Studies

The development of novel electron/hole conducting materials for perovskite photovoltaics [4,5,27] including molecular conductors to replace *spiro*-OMeTAD is surely one of the leading challenges in the field. The UV-Vis absorption spectra of the investigated imine were measured in chloroform and chlorobenzene solutions and in thin film. The absorption spectra of the bTAThDaz in chloroform solution display three partially overlapped signals with maxima at 247 nm, 297 nm and 375 nm. They are observed in the same range as for *spiro*-OMeTAD (see Figure S6). Figure S7 shows the optical spectrum of bTAThDaz in chlorobenzene solution at different concentrations. The molar extinction coefficients equal ε_297_ = 154,000 M^−1^∙cm^−1^ and ε_370_ = 149,000 M^−1^∙cm^−1^. They are about two times larger as compared to the extinction coefficients of *spiro*-OMeTAD in the same solvent. The corresponding maxima of are blue-shifted by ca. 20 nm as referenced to those of *spiro*-OMeTAD [28]. Honestly, the larger optical absorbance of bTAThDaz is an issue for inverted perovskite solar cell architecture (p-i-n), but not for the regular one (n-i-p).

**Figure 4:**
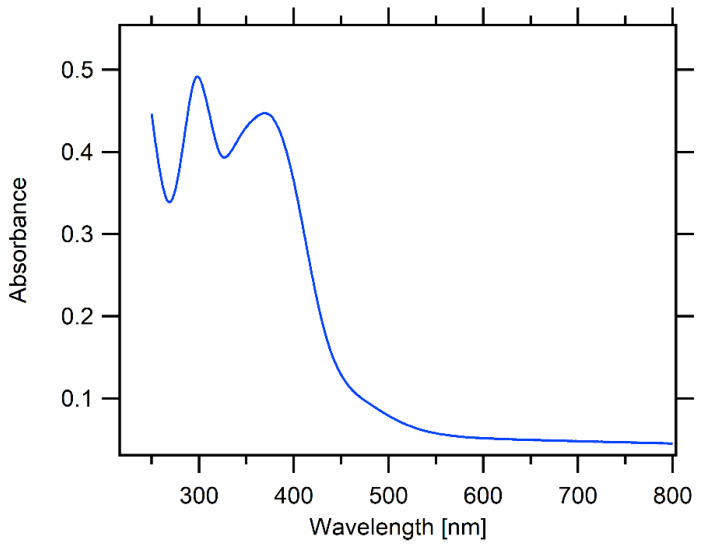
The optical (UV-Vis) spectrum of bTAThDaz thin film on quartz substrate.

The optical spectrum of thin-film of bTAThDaz is shown in Figure 4. Obviously, the spectrum of solution (Figure S6) is well reproduced on the thin film. Notably, the positions of both peaks’ maxima (297 and 370 nm) are identical in solid film as well as in the chlorobenzene solution, confirming that the electronic structure of bTAThDaz is unchanged. The optical band gap (*E*_g_^opt^ in eV) can be estimated from the equation: *E*_g_^opt^ = 1240/λ_edge_ where λ_edge_ is the wavelength (in nm) of the absorption edge [29]. Our optical spectra give *E*_g_^opt^ of 2.9 eV for *spiro*-OMeTAD (matching well the literature values) and 2.4–2.6 eV for bTAThDaz. (In the latter case, the uncertainty is caused by the broad absorption tail near the edge).

The electrochemical oxidation and reduction onset potentials were used to determine the HOMO and LUMO energies (ionization potentials (IP) and electron affinities) of the materials in solution, assuming the IP of the reference ferrocene to be −5.1 eV [30]. The obtained values of the HOMO-LUMO levels together with the electrochemical energy band gap (Eg^CV^) are presented in Table 1 and Figure S8. 

The cyclic voltammogram of bTAThDaz demonstrated three oxidation processes with maxima at 0.05, 0.29 and 0.82 V and only one reduction offset at −2.05 V. The lowest original cathodic peak came from thiadiazole, due to its strong aromaticity [31]. For bTAThDaz there is no difference between the thiadiazole ring and imine signals which suggest that the aromaticity of 1,2,4-thiadiazole ring expands on neighboring imine bonds. To summarize: 

Though peak in CV is very broad the LUMO tail of the reference *spiro*-OMeTAD in ER-EIS is well defined and sufficiently (0.4 V) far from −2.9 V (reduction of ACN in the glove box with Ar protective atmosphere). 

The LUMO position of −2.2 eV recalculated vs vacuum level is in agreement with commonly reported value. 

The value of −2.36 V vs Ag/AgCl observed for bTAThDaz is even 0.5 V above the −2.9 V, which warrants reliability of the presented data.

The calculation of LUMO from Eg^opt^ and HOMO CV gives value ranging −2.59 V −2.79 V, which is in good agreement with LUMO data obtained from CV experiment (−2.52 V). 

Moreover, the voltammogram for acetonitrile with Bu_4_NPF_6_ showed the reduction offset at −2.27 V vs. Ag/AgCl, which is 0.22 V above value for bTAThDaz in solution, hence the influence of the solvent reduction process does not overlap with reduction of the studied imine.

**Figure 5:**
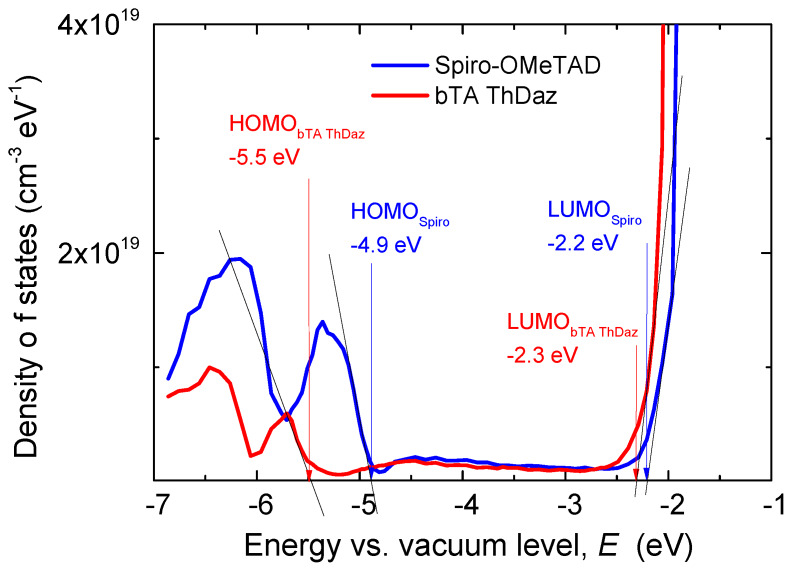
ER-EIS spectra in linear scale measured on imine bTaThDaz and *spiro*-OMeTAD thin films in acetonitrile with 0.1 M TBAF_6_. The frontier orbitals (HOMO and LUMO) were determined from the intersect of the of band tail extrapolation to the energy scale.

The measured ER-EIS spectra of the bTAThDaz film along with *spiro*-OMeTAD film are shown in Figure 5. The linear scale of the ER-EIS spectra was used to determine the HOMO and LUMO positions in such a way that the extrapolation of band tails intersects the energy scale [32]. Both the HOMO and LUMO positions are summarized in Table 1 together with the respective transport gaps. Unlike CV, the ER-EIS technique eliminates the parasitic currents and improves the sensitivity by several orders of magnitude. It is worth noting that the absorbance spectra of both films show slightly different absorption edges (Figure 4 and Figure S6) [28] confirming smaller band gap for the imine bTAThDaz film. In general, we can note reasonable agreement of the optical band gap *E*_g_^opt^ (see above) and Eg^CV^ measured in solution. Values from ER-EIS on thin films are similarly consistent for *spiro*-OMeTAD, but not for bTAThDaz. A difference between optical and transport band gaps of thin film is common characteristic of organic semiconductors. The optical band gap characterizes bound electron-hole pair (exciton). The transport band gap, measured with electrochemical techniques, can be larger because the exciton binding energy has to be overcome to achieve charge transport of free polarons [33], which is the case of the bTAThDaz film. 

### 3.3. Electrical Properties of Imine and Simple Devices together with Surface Morphology Checked by SEM and Thermographic Camera

**Figure 6:**
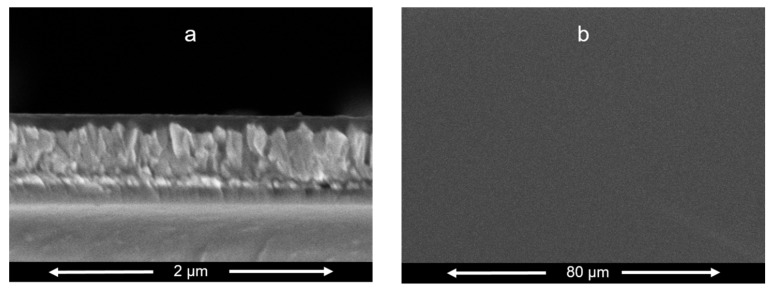
SEM images of the bTAThDaz thin film of FTO substrate. The chart (**a**) shows a cross-section view of the cut. The chart (**b**) shows a top view of the same film.

The surface conductivity of our thin film on quartz substrate (measured by 4-point probe) was poorly reproducible in the range of σ ≈ (10^−8^–10^−10^) S/cm. This conductivity is lower than that of *spiro*-OMeTAD (in the undoped state) [32]. The latter is, however known to increase by orders of magnitude upon doping, e.g., with LiTFSI [31] or Cu(II) pyridine complexes [34]. The conductivity of naturally doped CuSCN (which is another promising hole-conductor for perovskite photovoltaics) is ca. 10^−4^ S/cm but the experimental values significantly depend on the sample environment [5]. The two-point conductivity measured across the film (Au/bTAThDaz/Au) was by several orders of magnitude better (see also below), but again poorly reproducible, perhaps due to inhomogeneity in the film (see Figure 6 and Figure S2). Obviously, doping of bTAThDaz is a challenge for future research.

Our SEM images (Figure 6) show a reasonably compact morphology, and film thickness of ca 50–100 nm for the optimized films which were fabricated by spin-coating from the long-time sonicated solution (see Experimental Section). Figure S2 in Supplementary Materials shows the example case of non-optimized (heterogeneous) film containing micron-sized islands. Their successful removal is documented by Figure 6b.

To investigate defects in organic photovoltaics, thermal imaging as a fast, simple and cheap method for finding defects was applied [23,35,36,37,38].

**Figure 7:**
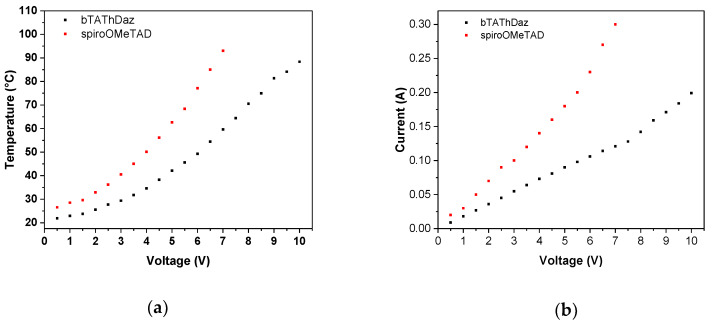

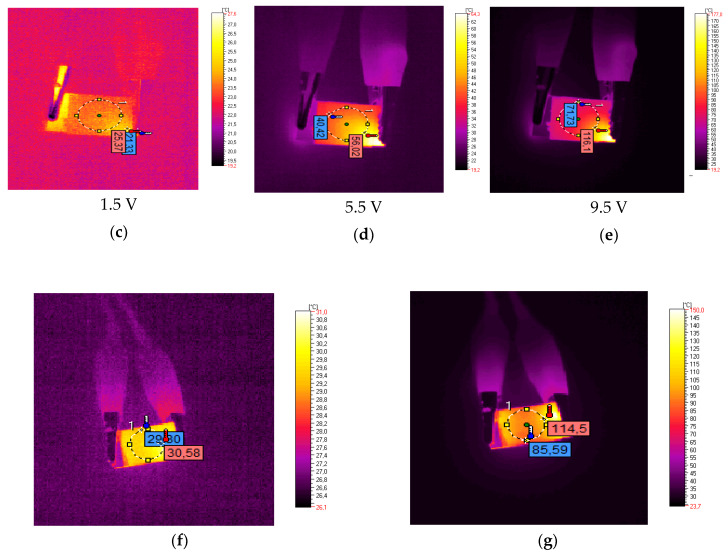
The correlation of temperature **(a**) and current flow (**b**) versus applied potential for constructed devices containing bTAThDaz and *spiro*-OMeTAD, as organic layer, together with IR images recorded at 1.5 V (**c**), 5.5 V (**d**) and 9.5 V (**e**) for bTAThDaz, and at 1.5 V (**f**) and 7 V (**g**) for *spiro*-OMeTAD, obtained for constructed devices with architecture glass/ITO/organic layer/Ag/ITO/glass.

Thermal imaging was carried out on devices made of spin-coated layer of bTAThDaz on ITO conductive glass and with silver contact with following architecture: ITO/bTAThDaz/Ag/ITO. Figure 7 shows the obtained data. The thermal images prove reasonable homogeneity of our organic layer, which is consisted with SEM images (Figure 6). Also, it was noticed that the organic layer behaved as conductor exhibiting the ohmic increase of current over increment of potential. Within the measured range for bTAThDaz a degradation was observed, when the temperature reached above 73 °C and 85 °C, respectively (see Figure 7). The resistance on 1 cm^2^ layer was similar and was equal 57.5 Ω, what is consistent with above mentioned conductivity measurements. 

In the case of bTAThDaz, the influence of the thickness of the sample on the electrical properties was also evaluated and it was observed that the thinner layer (approx. 20 nm) has a higher resistance of ca. 6 Ω compared to the sample with estimated thickness of 40 nm. It might be a result of different molecular rearrangement within the layer.

Additionally, Figure 7 shows defects in a device with *spiro*-OMeTAD investigated by thermographic camera. The thermal imaging for the device containing *spiro*-OMeTAD gave mainly homogenous-like distribution of the temperature with noticeable heat centers on the edges close to both electrodes, differently from devices containing bTAThDaz, where the highest temperature was observed only close to positive electrode or in the central part. Electrical behavior confirms conductive behavior of the *spiro*-OMeTAD layers. Moreover, comparing the electrical properties of layers prepared from *spiro*-OMeTAD with those prepared with new composition of active layers, one can see that in the case of commercial compound the current values are higher giving resistance of 24.05 Ω, almost a half of the value obtained for bTAThDaz. The largest disadvantage of the commercial material are the local heat maxima, located close to both metallic electrodes.

**Figure 8:**
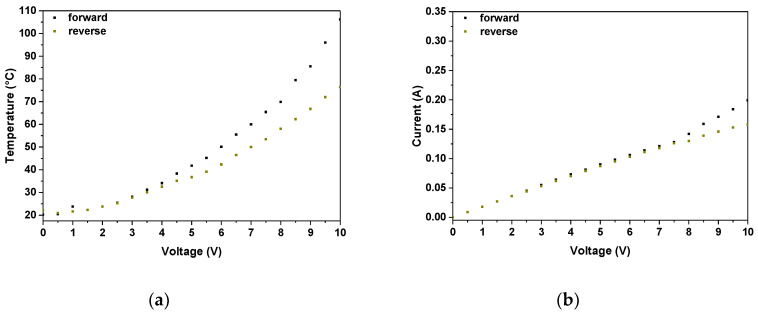
The correlation of temperature (**a**) and current flow (**b**) versus applied potential in forward and reverse mode for devices with the architecture glass/ITO/bTAThDaz/Ag/ITO/glass.

Furthermore, an additional study correlating electrical and thermal response of constructed devices with the direction of set potential was carried out. For this purpose, thermal imaging and current flow were recorded for bTAThDaz in forward and reverse current, similarly to previously mentioned experiment changing the polarization of the electrodes. The studies evidence that the thermal and electrical behavior of the imine is similar to the results presented in the previous section (Figure 8) regardless the direction of current flow. All small distortions could be an effect of superficial thermal reflections from the thermal maxima since the current flow lied in nearly exact range.

### 3.4. Perovskite Solar Cells with bTAThDaz

**Figure 9:**
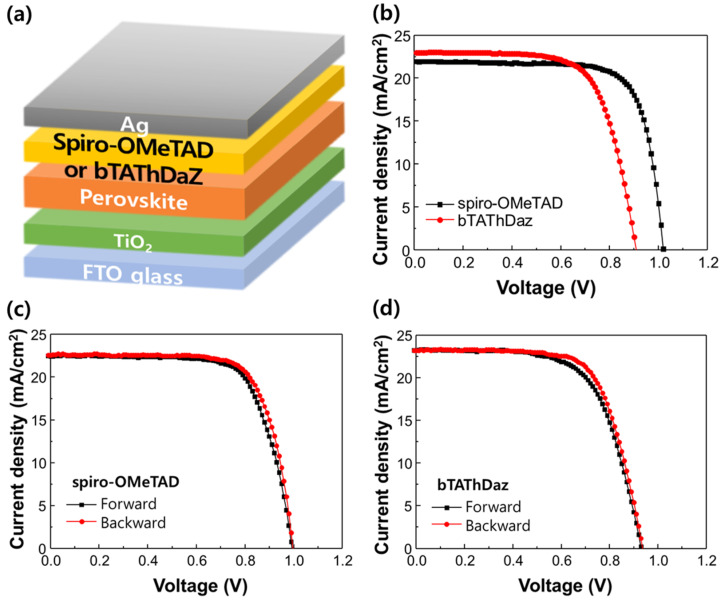
(**a**) Device architecture of perovskite solar cell used for this study. (**b**) Average current density-voltage curves of perovskite solar cells used *spiro*-OMeTAD and bTAThDaz as hole transport layer (12 samples). Current-voltage hysteresis curves of perovskite solar cells used (**c**) *spiro*-OMeTAD and (**d**) bTAThDaz measured starting with backward scan and continuing with forward scan.

Finally, we tested the bTAThDaz imine as HTM in perovskite solar cell. The photovoltaic performances of the perovskite solar cells (PSCs) using *spiro*-OMeTAD and bTAThDaz as hole transport materials are provided in Figure 9b. The characteristics of PSCs are summarized in Table 2. The open circuit voltage (V_oc_), short-circuit current (J_sc_), fill factor (FF), and power conversion efficiency (PCE) of the *spiro*-OMeTAD based PSCs are 1.02 V, 21.94 mA/cm^2^, 0.75, and 16.88%, respectively. The bTAThDaz hole transport layer gives a PCE of 14.37% with J_sc_ of 22.96 mA/cm^2^, V_oc_ of 0.90 V, and FF of 0.70. The conversion efficiency of bTAThDaz based PSCs was lower than that of *spiro*-OMeTAD based PSCs, but the J_sc_ was higher. 

With these devices, we compared the hysteretic behaviour with *spiro*-OMeTAD and bTAThDaz under standard AM 15.G sunlight in Figure 9c,d. The average efficiency scanned in reverse and forward bias directions of *spiro*-OMeTAD based PSCs was 16.29% with Voc of 0.99 V, Jsc of 22.51 mA/cm^2^, and FF of 0.73, that of bTAThDaz based PSCs was 14.47% with Voc of 0.93 V, Jsc of 23.22 mA/cm^2^, and FF of 0.67. It means that bTATaDaz can be used as hole transport material in the PSCs under optimized conditions.

The higher J_sc_ on the PSC using bTATaDaz as the hole transporter is counter-intuitive considering the found smaller conductivity of bTATaDaz (see above). Hence, this difference must be caused by some other effect(s), such as more efficient hole extraction at the interface CH_3_NH_3_PbI_3_/bTATaDaz as compared to the interface CH_3_NH_3_PbI_3_/*spiro*-OMeTAD. (At this stage of our research, we must keep this questions open, but presumably, the measurement of external quantum efficiency (IPCE) vs. wavelength could help to address this issue). On the other hand, the small value of V_oc_ of bTAThDaz based PSCs can be ascribed to the decrease of electron lifetime in the perovskite layer.

## 4. Conclusions 

Selected electrochemical properties are presented for an imine based on TPA units and thiadiazole moieties as prospective, air- and thermally stable candidate of organic layer for perovskite solar cells. Our study showed that bTAThDAz can be used as a component of HTM. 

The synthesis of imine was performed in an one-step reaction in solution followed by simple purification which significantly lowers the production cost of pure product. Additionally, the simplicity of the process allows recovery of used solvent and reuse again in other processes. The absorption properties in the UV-Vis range of bTAThDaz are almost identical with the properties of *spiro*-OMeTAD, which is a promising characteristic. Moreover, the investigated imine is stable in argon and air atmosphere and exhibited 5% of weight loss at 156 °C. As for the layers based on bTAThDaz do not suffer from current direction changes, that makes this layer conductive in both directions equally and the imine does not form secondary bonds and is stable up to 180 °C. We have constructed perovskite solar cells with imine as HTM that showed PCE = 14.4%. Summarizing, constructed devices with new HTM are time stable and exhibit negligible hysteresis effect. 

Taking into account all the reported results, the designed and synthesized bTAThDaz compound appear to be a promising material for use as a conductive organic layer in perovskite solar cell however additional modifications including for example doping of imine are required to improve charge transport properties of the investigated compound. The good photovoltaic performance of bTAThDaz means that bTAThDAz can be used as hole transport material in perovskite solar cells. In this work the highest value of PCE was obtained for PSCs based on imine as HTM. In comparison with results presented in [1] where PCE = 11.0% was found for their device with EDOT-OMeTPA, we increased the efficiency of PSCs with our imine (bTAThDaz) by more than 30% and received PCE = 14.4%. Furthermore, our HTM material is thermally and air stable with very small hysteresis effect. 

## Figures and Tables

**Table 1:** Electrochemical (CV) in solution, energy-resolved electrochemical impedance spectroscopy (ER-EIS) in thin film and optical (UV-Vis) properties of imine and *spiro*-OMeTAD in solution.

Code	E_ox_^onset^ (V)	E_red_^offset^ (V)	HOMO (eV)		LUMO (eV)		Eg (eV)		λ_abs_ [nm] in CHCl_3_
			CV	ER-EIS	CV	ER-EIS	CV	ER-EIS	
bTAThDaz	0.74	−2.05	−5.19	−5.5	−2.52	−2.3	2.67	3.2	247, 297, 375
*spiro*-OMeTAD	0.71	−2.23	−5.16	−4.9	−2.34	−2.2	2.82	2.7	247, 308, 388

**Table 2:** Photovoltaic parameters of investigated perovskite solar cells with various HTM.

HTM		J_sc_(mAcm^2^)	V_oc_(V)	Efficiency(%)	FF
Spiro-OMeTAD	Average (12 samples)	21.94 ± 1.0	1.02 ± 0.02	16.88 ± 1.0	0.75 ± 0.05
	Backward	22.57	0.99	16.54	0.74
	Forward	22.45	0.99	16.05	0.72
bTAThDaz	Average (12 samples)	22.96 ± 1.2	0.90 ± 0.02	14.37 ± 0.8	0.70 ± 0.06
	Backward	23.22	0.93	14.89	0.6
	Forward	23.23	0.93	14.05	0.65

## Supplementary Materials

The following are available online at https://www.mdpi.com/1996-1944/13/11/2440/s1, Figure S1: Graphical representation of the experimental timeline of thermal imaging experiment. Figure S2: SEM image of a FTO-supported thin film, which was fabricated from 70 mM chlorobenzene solution. The solution was not subjected to prolonged sonication. The film shows microheterogeneity caused by undissolved particles of the solid precursor. Figure S3: XPS high resolution spectra of the (a) C1s, (b) N1s and (c) S2p together with XPS survey scan of bTAthDaz. Figure S4: (a) Powder X-ray diffractogram of bTAthDaz. The pattern of a blank sample holder is shown too. (b) DSC curve of bTAthDaz registered at heating. Figure S5: Room-temperature infrared spectrum of bTAThDaz and assignment of the vibrational modes in the range 400–1750 cm^−1^. Figure S6: Comparative UV-Vis spectra of imine bTAThDaz and *spiro*-OMeTAD in chloroform solution. Figure S7: The UV-Vis spectrum of bTAThDaz in chlorobenzene solution and reference spectrum of pure solvent (dashed line). Two different concentrations of solution were tested as marked in the annotation. Quartz optical cell, 1 cm length. Figure S8: Cyclic voltammograms of bTAThDaz in acetonitrile solution containing Bu_4_NPF_6_ as electrolyte solution. Blank sample was done for Bu_4_NPF_6_ in acetonitrile. Table S1: Surface chemical composition of bTAThDaz obtained from XPS.
