# Supplementary material for: Selected Electrochemical Properties of 4,4’-((1E,1’E)-((1,2,4-Thiadiazole-3,5-diyl)bis(azaneylylidene))bis(methaneylylidene))bis(N,N-di-p-tolylaniline) towards Perovskite Solar Cells with 14.4% Efficiency
Source: Materials (Basel). 2020 May 27;13(11):2440. doi: 10.3390/ma13112440 (PMC7321367; doi:10.3390/ma13112440)
Supplement: Supplementary file 1 [file materials-13-02440-s001.pdf]

Experimental

Synthesis of imines 4,4'-((1E,1'E)-((1,2,4-thiadiazole-3,5-

diyl)bis(azaneylylidene))bis(methaneylylidene)) bis(N,N-di-p-tolylaniline) (bTAThDaz) [20,21]

The imine was obtained using a one-step high temperature condensation reaction under reflux equipped with anhydrous $CaSO_4$ as the water trap. A single-neck flask with a magnetic stir bar was charged with 4-(di-p-tolylamino)benzaldehyde) (2 mmol), 1,2,4-thiadiazole-3,5-diamine (1 mmol), *p*-toluenesulfonic acid (PTS) and 12 mL of DMA. The reaction mixture was stirred for 24 h at 160 °C in an oil bath. The raw imine was precipitated in water, and collected by filtration. The solid was washed with ethanol, acetone, and recrystalized from acetone:hexane. The final the imine was dried overnight at 80 °C.

bTAThDaz: Red-brown powder, Yield: 45%. ^{1}H NMR (400 MHz, $CDCl_3$), δ [ppm]: 8.31 (2H, d, -HC=N-); 7.65-7.55 (8H, m, arom. -Ph-); 7.4-7.0 (16 H, arom. -Ph-); 2.35 (12H, t, $-CH_3$).

FTIR (cm^{-1}): 3025w $\nu(CH)_{ar}$, 2920m $\nu_{as}(CH_3)$, 2855w $\nu_s(CH_3)$, 2217m , 1617w ν(HC=N), 1597m $\nu(C=C)_{ar}$, 1506m δ(CCH), 1387m δ(CCH), 1320m ν(C-C), 1294br ν(C=N), 1173m δ(CCH), 814s $\pi(CCH)_{ar}$, 722m $\omega(CCH)_{ar}$, 567m δ(CNC), 517s γ(CCC) (w – weak, m – medium, s-strong, br – broad,ν – stretching; δ – bending in-plane, π - bending out-of-plane). m.p. 153.8 °C.

The sample was characterized with ^{1}H NMR, using deuterated chloroform ($CDCl_3$) as a solvent with a Jeol ECZ-400 S spectrometer (^{1}H - 400 MHz) with delay time 5 s. Measurements were carried out at room temperature on 10-15% (w/v) sample solutions.

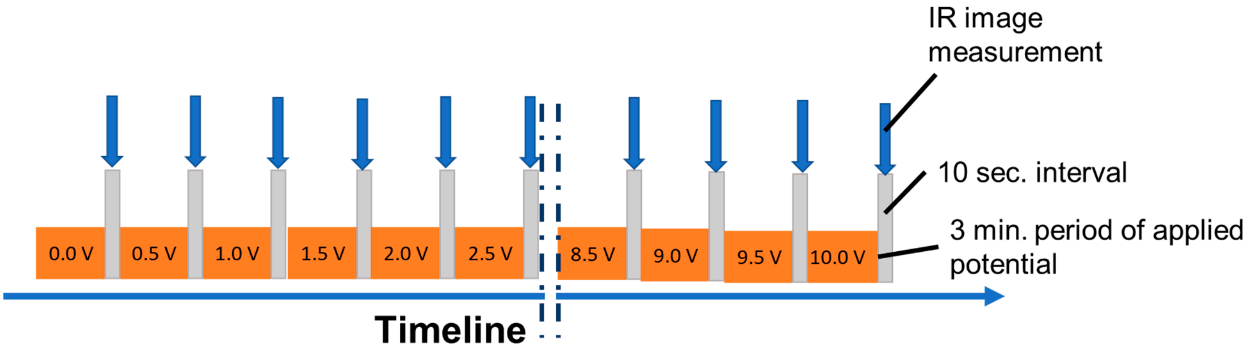

Figure S1. Graphical representation of the experimental timeline of thermal imaging experiment.

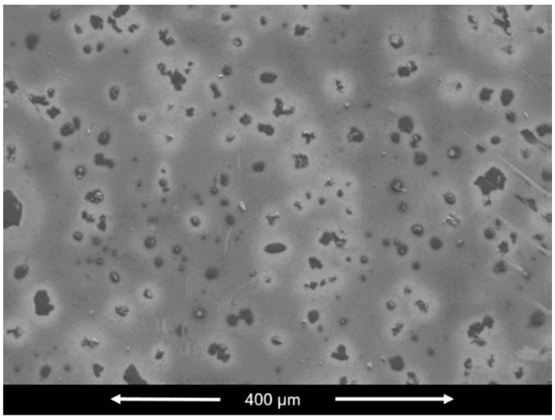

Figure S2. SEM image of a FTO-supported thin film, which was fabricated from 70 mM chlorobenzene solution. The solution was not subjected to prolonged sonication. The film shows microheterogeneity caused by undissolved particles of the solid precursor.

Table S1. Surface chemical composition of bTAThDaz obtained from XPS.

**Element (Overall Content, at.%)**	**State**	$E_{BE, max}$, **eV**	**at. %**
C1s (76.0)	sp^2	284.2	48.1
	sp^3	284.9	17.6
	C-N	285.6	4.4
	C=N	287.4	4.2
	π-π*	291.0	1.7
O1s (6.7)	C=O	531.0	1.5
	$C\text{-}O_{al}$	531.9	4.8
	$C\text{-}O_{ar}$	533.4	0.4
N1s (12.2)	Imine	398.3	6.5
	Amine	399.5	4.9
	N^+	400.4	0.8
S2p (1.3)	S^{2-}	161.8	0.5
	-S-	164.0	0.5
	SO_4	167.5	0.3
Si2p (3.8)	SiO_x	101.8	3.8

Apparent surface chemical composition of prepared bTAThDaz is showed in Table 1. Typical peaks of bTAThDaz were detected, C1s (76.1 at.%), N1s (12.2 at%) and S2p (1.3 at.%). Besides these peaks also oxygen was present (O1s, 6.7 at.%), mostly coming from the contamination by some siloxanes (Si2p signal at ca 101.8 eV, 3.8 at.%). Aromatic carbon is confirmed by the signal of sp^2 carbon (centered at ~284.1 eV) and π–π* shake-up (~291.0 eV), the structure of bTAThDaz is confirmed by the presence of amine (C-N at ~285.6 eV) and imine (C=N at ~287.4 eV) [1]. This shows also signal from nitrogen with three chemical states one assigned to imine (–N= at ~398.3 eV), amine (C-N at ~ 399.5 eV) and some oxidized or quaternized nitrogen at ~ 400.4 eV. Sulphur is interestingly also in three oxidation states one at ~ 164.0 eV corresponding to -S- in the structure of bTAThDaz and then one at higher binding energies (at ~ 167.5 eV) coming from some surface oxidation and one at lower energies (at ~ 161.8 eV) coming from some reduced form of sulphur. This signal at lower binding energy might be also some thiolate ($-S^-$) as a consequence of cleavage of C-S bonds.

Beamson, G., Briggs, D., 1992, High Resolution XPS of Organic Polymers - The Scienta ESCA300 Database. Wiley Interscience.

a)

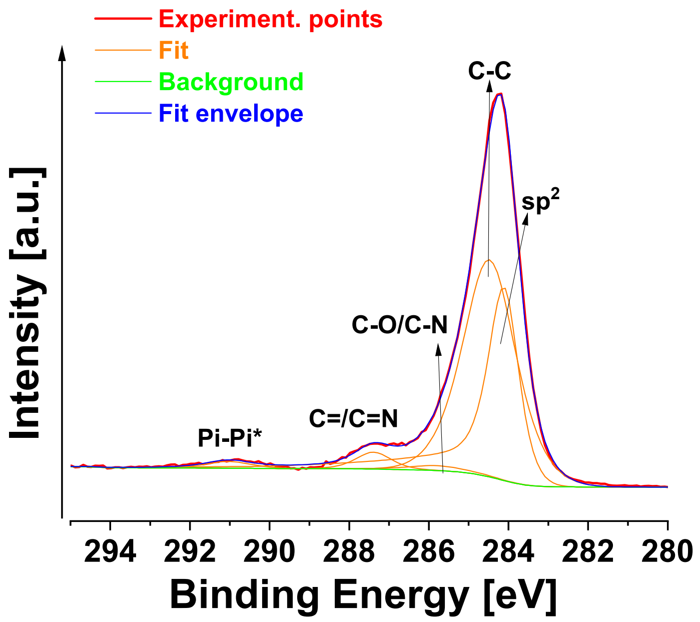

Experiment. points
Fit
Background
Fit envelope
C-C
sp2
C-O/C-N
C=/C=N
Pi-Pi*
Intensity [a.u.]
294
292
290
288
286
284
282
280
Binding Energy [eV]

b)

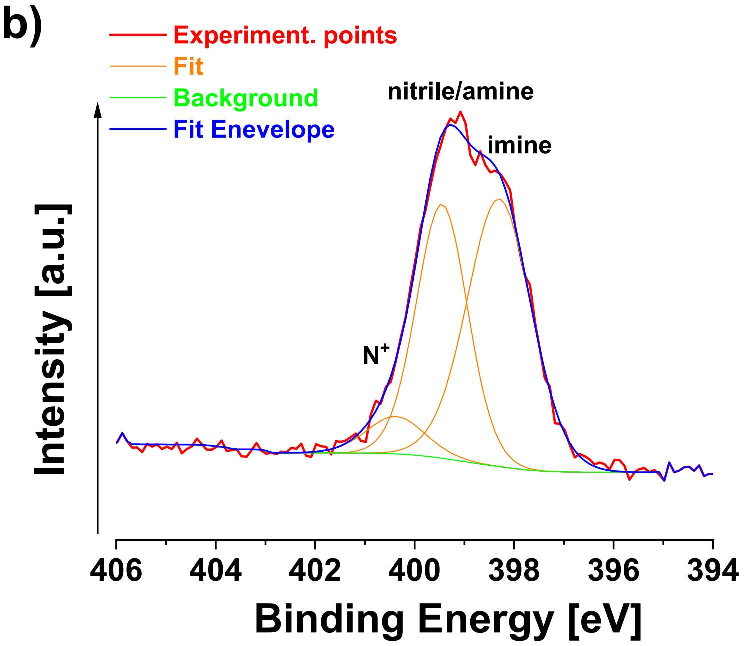

Experiment. points
Fit
Background
Fit Enevelope
nitrile/amine
imine
N+
Intensity [a.u.]
406
404
402
400
398
396
394
Binding Energy [eV]

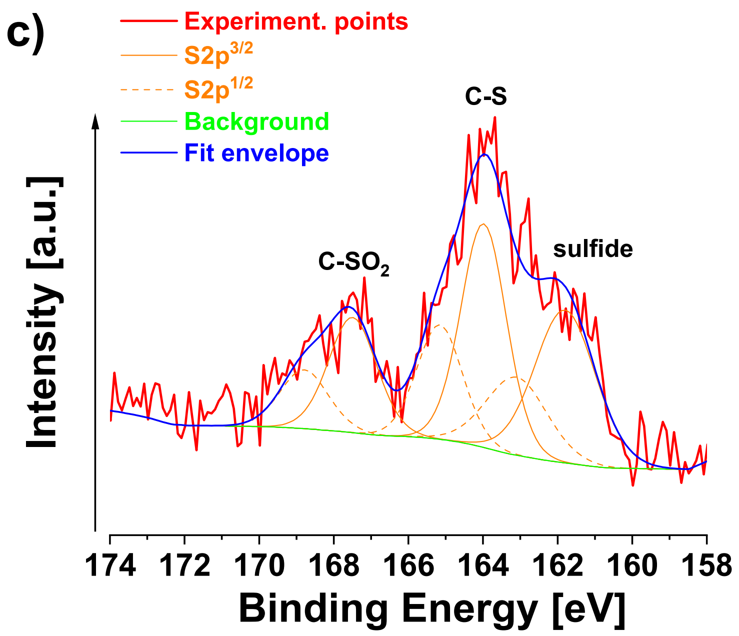

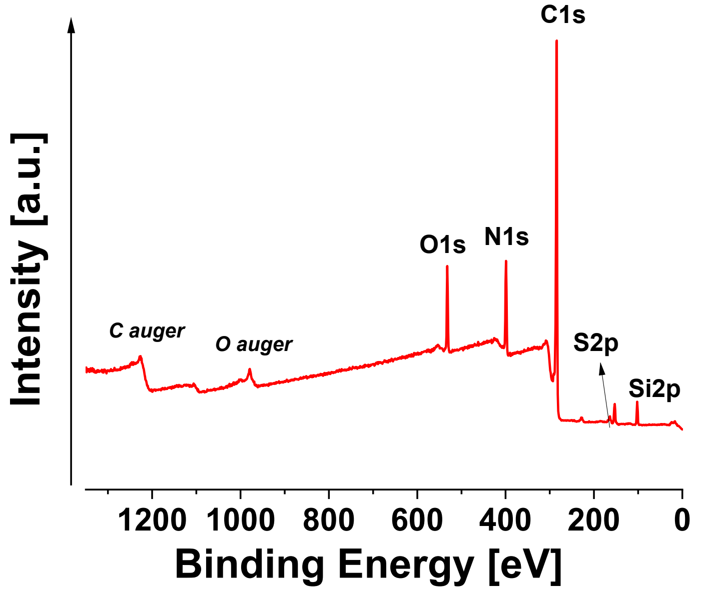

Figure S3. XPS high resolution spectra of the (**a**) C1s, (**b**) N1s and (**c**) S2p together with XPS survey scan of bTAthDaz.

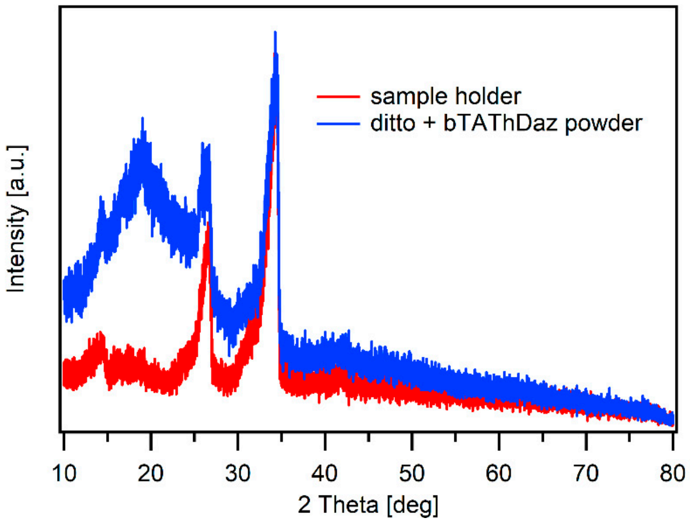

(a)

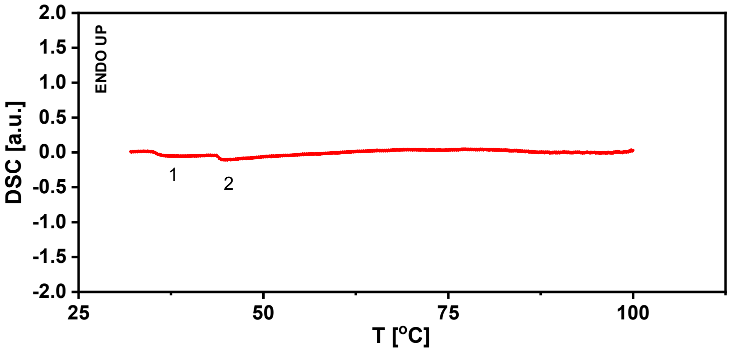

(b)

Figure S4. (**a**) Powder X-ray diffractogram of bTAthDaz. The pattern of a blank sample holder is shown too. (**b**) DSC curve of bTAthDaz registered at heating.

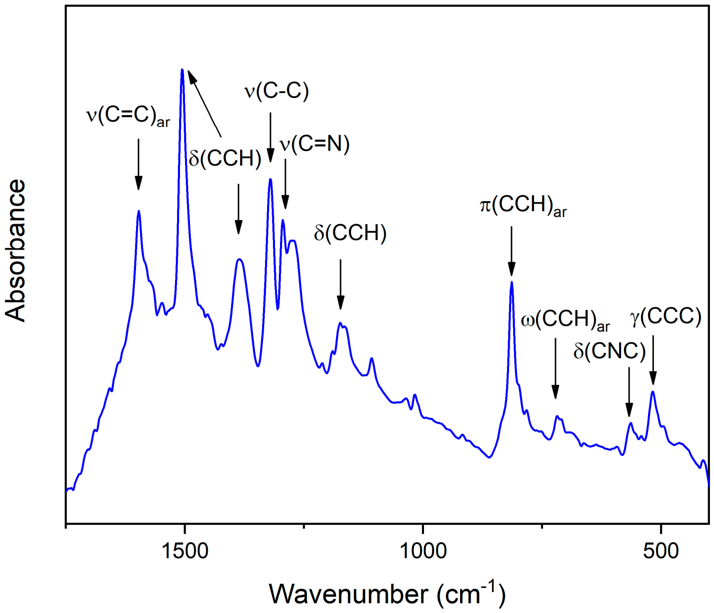

Figure S5. Room-temperature infrared spectrum of bTAThDaz and assignment of the vibrational modes in the range 400–1750 cm^{-1}.

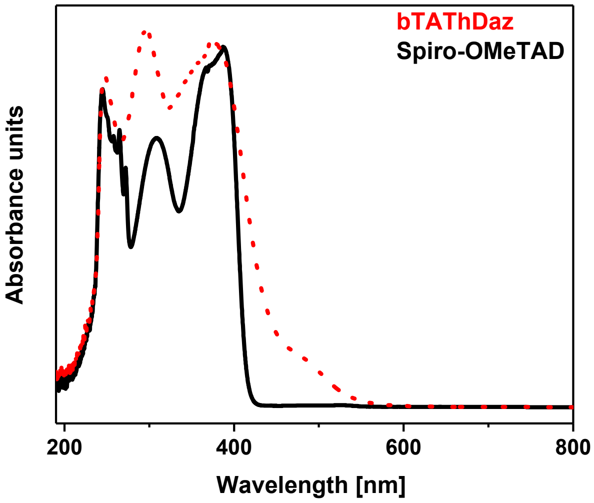

Figure S6. Comparative UV-Vis spectra of imine bTAThDaz and spiro-OMeTAD in chloroform solution.

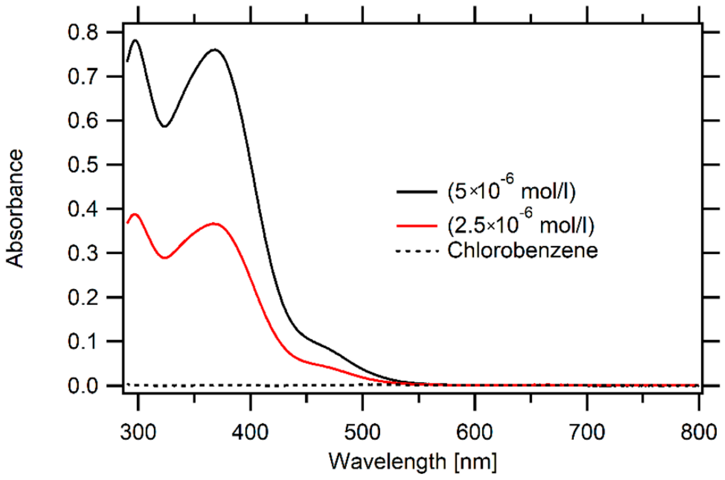

Figure S7. The UV-Vis spectrum of bTAThDaz in chlorobenzene solution and reference spectrum of pure solvent (dashed line). Two different concentrations of solution were tested as marked in the annotation. Quartz optical cell, 1 cm length.

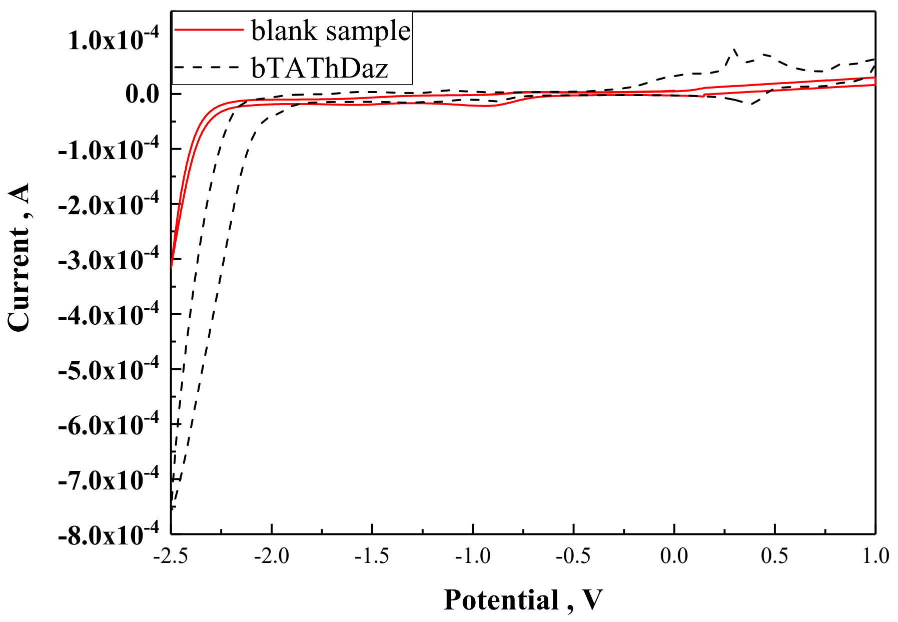

Figure S8. Cyclic voltammograms of bTAThDaz in acetonitrile solution containing Bu4NPF6 as electrolyte solution. Blank sample was done for Bu4NPF6 in acetonitrile.
